# Supplementary material for: Association of TLR-4 and TLR-9 Polymorphisms with HPV Infection and Cervical Dysplasia in Hispanic Women
Source: Cancers (Basel). 2025 Nov 27;17(23):3795. doi: 10.3390/cancers17233795 (PMC12691510; doi:10.3390/cancers17233795)
Supplement: Supplementary file 1 [file cancers-17-03795-s001.zip › Supplementary Tables S1–S4.pdf]

Supplementary Table S1. Power Calculations

Power Calculations for Cervical Phenotype Groups:

Cervical Phenotypes groups	Effect Size (Cramer's V)	Degrees of Freedom	Alpha Level	Calculated Power
TLR-4	0.135	6	0.05	0.46
TLR-9	0.130	9	0.05	0.53

With the effect size obtained from the results of this study, we would need approximately 400 patients to achieve a power of 80% using an alpha level of 0.05.

Power Calculations for HPV positivity Groups:

Cervical Phenotypes groups	Effect Size (Cramer's V)	Alpha Level	Degrees of Freedom	Calculated Power
TLR-4	0.118	0.05	2	0.28
TLR-9	0.174	0.05	3	0.50

Supplementary Table S2. Overall percentages of HPV vaccination

HPV vaccination	N (%)
No	140 (72.54)
Yes	25 (12.95)
Don't Know	19 (9.84)
Missing	9 (4.66)

Supplementary Table S3*: RsID, primers sequences, and thermal conditions for the PCR reaction.*

**Gene**	**rsID**	**Primers (5'-3')**	**Thermal conditions**	**Amplicon Size (bp)**
TLR4	rs4986790	F: GATTAGCATACTTAGACTACTACCTCCATG<br>R: GATCAACTTCTGAAAAAGCATTCCCAC	(95°-5')1[1]<br>(94°-40'',<br>55°-40'',<br>72°-60'')36[2]<br>(72°-10')1[3]	249
	rs10759931	F: ATAACCTCAGTGGGCTCTGG<br>R: ATGTTCTGGCATCTGGGAAG	(94°-5')1<br>(94°-40'',<br>58°-45'',<br>72°-40'')35<br>(72°-10')1	241
	rs11536889	F: ACAAGTGATGTTTGATGGAC<br>R: GCCATTCTACCTGGTATAAG	(94°-6')1<br>(94°-60'',<br>55°-60'',<br>72°-2')35<br>(72°-10')1	361
	rs1927911	F: TCACTTTGCTCAAGGGTCAA<br>R: AAACCTGCATGCTCTGCAC	(94°-5')1<br>(94°-40'',<br>58°-45'',<br>72°-40'')35<br>(72°-10')1	203
TLR9	rs187084	F: TCCCAGCAGCAACAATTCATTA<br>R: CTGCTTGCAGTTGACTGTGT	(95°-5')1<br>(95°-40'',<br>60°-40'',<br>72°-60'')36<br>(72°-10')1	499
	rs5743836	F: ATGGGAGCAGAGACATAATGGA<br>R: CTGCTTGCACTTGACTGTGT	(95°-5')1<br>(94°-40'',<br>62°-40'',<br>72°-60'')35<br>(72°-10')1	135
	rs352140	F: AAGCTGGACCTCTACCACGA<br>R: TTGGCTGTGGATGTTGTT	(95°-5')1<br>(94°-45'',<br>56°-60'',<br>72°-30'')35<br>(72°-10')1	177
	**rs352139**<br><br>**AS-PCR**	AFP: AAGTGGAGTGGGTGGAGGTA<br>GFP: GTGGAGTGGGTGGAGGTG<br>R: CAAGGAAAGGCTGGTGACAT	(95°-5')1<br>(94°-60'',<br>64°-60'',<br>72°-60'')35<br>(72°-4')1	270

Note*: F (Forward), R(Reverse), Thermal conditions: Initial denaturation[1], cycles of (denaturation, annealing and extension)[2] and final step[3]. (X'=minutes, X''=seconds) Temperature in Celsius.

Supplementary Table S4*. Restriction enzymes for each reaction and incubation temperatures*.

Gene	rsID	Restriction enzyme	Incubation temperature (°C)	Separation
TLR4	rs4986790	NcoI	37	**15 % PAGE**
	rs10759931	KpnI	37	**2.5 % Agarose**
	rs11536889	EarI	37	**2.5 % Agarose**
	rs1927911	StyI	37	**15 % PAGE**
TLR9	rs187084	AflII	37	**2% Agarose**
	rs5743836	BstNI	60	**15 % PAGE**
	rs352140	BstUI	60	**12 % PAGE**
	rs352139	N/A AS-PCR	N/A	**2 % Agarose**
